# A Bifunctional-Modulated Conformal Li/Mn-Rich Layered Cathode for Fast-Charging, High Volumetric Density and Durable Li-Ion Full Cells

**DOI:** 10.1007/s40820-021-00643-1

**Published:** 2021-05-02

**Authors:** Zedong Zhao, Minqiang Sun, Tianqi Wu, Jiajia Zhang, Peng Wang, Long Zhang, Chongyang Yang, Chengxin Peng, Hongbin Lu

**Affiliations:** 1 State Key Laboratory of Molecular Engineering of Polymers, Department of Macromolecular Science, Collaborative Innovation Center of Polymers and Polymer Composites, Fudan University, 2005 Songhu Road, Shanghai, 200438 People’s Republic of China; 2 School of Materials Science & Engineering, University of Shanghai for Science and Technology, Shanghai, 200093 People’s Republic of China; 3 National Engineering Research Center for Supercapacitor for Vehicles, Shanghai Aowei Technology Development Co., Ltd, Shanghai, 201203 People’s Republic of China; 4 Key Laboratory of Advanced Energy Materials Chemistry (Ministry of Education), College of Chemistry, Nankai University, Tianjin, 300071 People’s Republic of China

**Keywords:** Lithium- and manganese-rich layered cathode, Semi-hollow microspheres, Volumetric energy density, Conformal structure, Full cell

## Abstract

Lithium- and manganese-rich (LMR) layered cathode materials hold the great promise in designing the next-generation high energy density lithium ion batteries. However, due to the severe surface phase transformation and structure collapse, stabilizing LMR to suppress capacity fade has been a critical challenge. Here, a bifunctional strategy that integrates the advantages of surface modification and structural design is proposed to address the above issues. A model compound Li_1.2_Mn_0.54_Ni_0.13_Co_0.13_O_2_ (MNC) with semi-hollow microsphere structure is synthesized, of which the surface is modified by surface-treated layer and graphene/carbon nanotube dual layers. The unique structure design enabled high tap density (2.1 g cm^−3^) and bidirectional ion diffusion pathways. The dual surface coatings covalent bonded with MNC via C-O-M linkage greatly improves charge transfer efficiency and mitigates electrode degradation. Owing to the synergistic effect, the obtained MNC cathode is highly conformal with durable structure integrity, exhibiting high volumetric energy density (2234 Wh L^−1^) and predominant capacitive behavior. The assembled full cell, with nanographite as the anode, reveals an energy density of 526.5 Wh kg^−1^, good rate performance (70.3% retention at 20 C) and long cycle life (1000 cycles). The strategy presented in this work may shed light on designing other high-performance energy devices.

**Supplementary Information:**

The online version contains supplementary material available at 10.1007/s40820-021-00643-1.

## Highlights

- A lithium- and manganese-rich (LMR) layered cathode with semi-hollow microsphere structure is synthesized, of which the unique structure design enabled high tap density (2.1 g cm^−3^) and bidirectional ion diffusion pathways
- The surface coatings covalent bonded with LMR via C-O-M linkage greatly improves charge transfer efficiency and mitigates surface degradation
- The LMR is highly conformal with durable structure integrity, exhibiting high volumetric energy density (2234 Wh L^−1^) and long cycling life (1000 cycles)

**Supplementary Information:**

The online version contains supplementary material available at 10.1007/s40820-021-00643-1.

## Graphical abstract

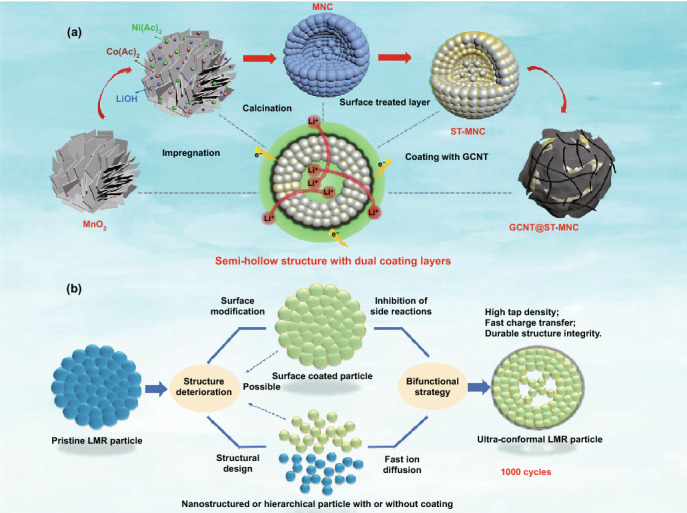

## Introduction

With the ever-growing global market of electric vehicles, the demand for longer driving range has posed a great challenge to the energy density of lithium-ion batteries (LIBs) [1]. Target energy density of reaching 500 Wh kg^−1^ is urgently needed [2, 3]. The key to solve the energy density of LIBs lies in the breakthrough of cathode material design [4]. The lithium- and manganese-rich (LMR) layered cathode materials xLi_2_MnO_3_·(1-x) LiMO_2_ (M = Ni, Co, Mn or combinations) with high reversible specific capacity over 250 mAh g^−1^ open a new opportunity for the next-generation LIBs [5–12]. Although promising, several issues still hinder their practical application, including low initial coulomb efficiency, poor rate/cycle performance, and severe voltage/capacity fade [5, 6, 10, 11]. Upon cycling, due to the surface structure engendering severe phase transformation from the layered to defect spinel, ionic conduction path is blocked, causing great voltage fade [9, 13]. More importantly, significant lattice expansion (14.25–14.4 Å) during repeated charge/discharge may lead to crack formation in the primary particles, and even collapse of the secondary particle structure [14–17]. Consequently, ion/electron pathways are damaged, and the capacity drops rapidly. In addition, it has been demonstrated that the difference in ion diffusion rates upon charging at high voltages can induce stress concentration in local regions, resulting in fast formation of dislocations and voltage fade [18]. Therefore, it is of great importance to construct a high-conformal structure to ensure durable and fast ion/electron pathways (IEPs) for deeply cycled LMR particle.

To address the structure deterioration issue, various strategies have been developed to stabilize the surface and enhance structural stability. Among them, surface modification has been found to be effective as it can not only inhibit the phase transition and side reactions with the electrolytes or hydrogen fluoride, but also provide ion or/and electron highways in some cases [19–26]. Ion conductive coating materials such as Al_2_O_3_ [21], AlF_3_ [23, 27], and phosphates [26] have been proved to facilitate ion diffusion and enhance the rate capability. Also, electron conductive materials such as carbon [22] and polypyrrole [25] have revealed the ability to accelerate charge transfer kinetics. This implies that constructing IEPs can combine the above two advantages. For example, the mixed Mg^2+^ pillar and LiMgPO_4_ modification layers are effective in suppressing the side reactions between LMR and HF generated by the high voltage-induced electrolyte decomposition [20]. The perovskite-type La_1-x_Sr_x_MnO_3-y_ material with enhanced IEP has shown good interface compatibility during long cycling [24]. These modifications hold promise in improving the rate performance and suppressing capacity/voltage fade. However, the typical structure of large secondary particles aggregated from closely packed smaller primary particles is not always favorable for fast ion diffusion. Both blocked ion channels and significant volume expansion may make LMR face the risk of structural collapse in long-term cycling.

This poses a practical challenge for the rational design and morphological control of LMR with desired ion diffusion capability and structure integrality [28–35]. A variety of morphological control strategies, including microrods [31], thin plates with specific crystal planes exposed [30], hollow [33], and porous structures [32] have been synthesized to improve the performance of LMR cathodes. Among them, nanostructured LMR cathodes possess increased reaction areas, shortened ion diffusion paths, and mitigated volume expansion, revealing improved gravimetric energy densities, despite at the cost of reduced tap densities or volumetric energy densities. In practical applications, however, it could be more desired to have micro-level spherical particles with a high tap density above 2 g cm^−3^, because they are more readily dispersed in solvent and beneficial to increase the volumetric energy density [35]. In this regard, hierarchical structures have increasingly become an attractive solution given their structural advantage in nano- and micro-scales [36, 37]. Mesopores [32] or nanopores [28] contribute to increase the contact area and promote ion conduction. For example, a 3D porous nano/micro LMR cathode with the tap density of 2.2 g cm^−3^ has shown good rate performance [28]. Nevertheless, it would be noted that more surface areas could also induce more side reactions [29]. Although some optimizing strategies such nanoscale coatings have been attempted to mitigate surface deterioration [38, 39], the issues such as cycling stability and voltage fade still need to be resolved.

**Fig. 1:**
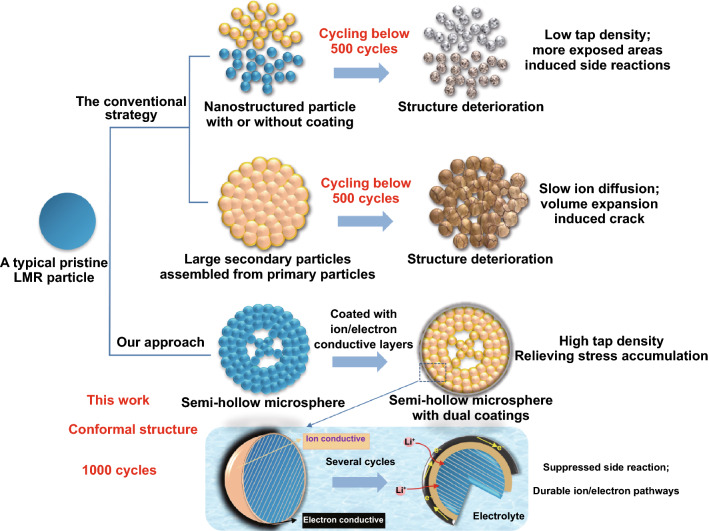
Schematic illustration of highlighting the high-conformal structure of the LMR particle. Compared with nanostructured or densely assembled LMR particle that may still suffer structure degradation, integrated advantages enabled by bifunctional strategy can be achieved

In this context, it is important to develop an optimal bifunctional modulation strategy by integrating surface modification and structural design for achieving high-performance LMR cathodes. Here, we report a semi-hollow Li_1.2_Mn_0.54_Ni_0.13_Co_0.13_O_2_ cathode (MNC) by dual coating layers of ion conductive surface treated layer (ST) and electron conductive graphene/carbon nanotubes (GCNT) (denoted as GCNT). Compared with the reported solid or completely hollow-structured spheres (see more details in Fig. S1), the semi-hollow structure is composed of a compact nanoparticle shell and loosely connected particles as the inner core. Such structure contributes to maximize space utilization per unit volume and realize a high tap density of 2.1 g cm^−3^. Internal cavities can also help buffer volume expansion and provide bidirectional (inside and outside) ion diffusion pathways. In addition, the ST and GCNT dual layers construct durable fast IEPs. Owing to the presence of the outer GCNT layer bonded on MNC through the C-O-M linkage, the cathode reveals excellent mechanical property and corrosion resistance and largely suppresses the side reactions with electrolyte. Structural stability of each single particle is thus reinforced. Such LMR cathodes possess high-conformal structure, lasting interphase stability (fast IEPs) and enhanced tap density (Fig. 1). When paired with a nanographite anode to assemble a full battery, the resulting LMR-based battery exhibits high volumetric energy density of 750 Wh L^−1^ (based on the total volume of cathode and anode), much improved energy density of 526.5 Wh kg^−1^, superior rate capability (0.1C-20C, 70% capacity retention) as well as long cycle life (91% retention after 1000 cycles).

## Experimental

### Synthesis of Li_1.2_Mn_0.54_Ni_0.13_Co_0.13_O_2_ (MNC) Semi-hollow Microspheres

The MnCO_3_ microspheres were prepared by a modified precipitation method. In brief, 1 mmol MnSO_4_·H_2_O and 10 mmol NH_4_HCO_3_ were separately dissolved in 70 mL distilled water. Then, 7 mL ethanol and NH_4_HCO_3_ solution were added to MnSO_4_·H_2_O solution in sequence under stirring. The mixed solution gradually became cloudy with milky-white color, indicative of initial formation of MnCO_3_, which was kept under stirring for 1 h at room temperature and then centrifuged, washed with deionized water and ethanol for three times. The as-obtained MnCO_3_ microspheres were freeze-dried for subsequent use. The porous MnO_2_ microspheres were acquired by sintering MnCO_3_ microspheres at 400 °C for 5 h in air with a heating rate of 1 °C min^−1^. To obtain Li_1.2_Mn_0.54_Ni_0.13_Co_0.13_O_2_ (MNC) semi-hollow microspheres, 12.5 mmol MnO_2_ microspheres, 3 mmol Ni (Ac)_2_·4H_2_O, 3 mmol Co(Ac)_2_·4H_2_O, 29 mmol LiOH·H_2_O were ultrasonic dispersed in 15 mL ethanol. The ethanol was evaporated slowly at room temperature under stirring. The resultant mixture was ground manually for 20 min and then was calcined at 800 °C for 20 h in air.

### Surface Treatment and Graphene/Carbon Nanotubes (GCNT) Coating of MNC

For surface treatment (ST), the MNC was modified using the completely ionized solution with lithium and cobalt sources. First, 0.3 mmol LiAc and 0.35 mmol Co (Ac)_2_·4H_2_O were dissolved in 25 mL ethanol. Next, 175 mg as-synthesized MNC was added into the above solution. The mixed solution was vigorously stirred at 70 °C until the ethanol completely evaporated. The mixture was ground to fine powder and finally calcined at 800 °C for 4 h in air to obtain ST-MNC.

10 mL graphene oxides/carbon nanotubes (1 mg mL^−1^ GOCNT) dispersion was dropwise added into 30 mL ST-MNC (5 mg mL^−1^) dispersion under ultrasonic and magnetic stirring for coating GOCNT onto the ST-MNC surface. Next, chemical treatment of the above dispersion was conducted with a typical condition of 0.1 wt% hydrazine solution for 1 h in 95 °C oil-bath followed by washing with deionized water. After freeze drying, the resulting product was annealed at 400 °C for 2 h to obtain GCNT@ST-MNC. The mass ratio of GCNT for GCNT@ST-MNC was about 4%.

### Characterization

The morphologies of the samples were observed by field-emission scanning electron microscopy (FESEM, Ultra 55) and high resolution transmission electron microscopy (HR-TEM, JEM-2100F, operating at 200 kV). X-ray diffraction (XRD) was carried out using an X’PertPRO (PANalytical) diffractometer operating at 40 kV and 40 mA with the monochromatic Cu Kα radiation (λ = 1.54 Å). Raman characterization was performed with a Raman spectrometer (XploRA, HORIBA Jobin Yvon) with a green excitation wavelength of 532 nm. Thermogravimetric analysis (TGA) was performed under air flow with a temperature ramp of 10 °C min^−1^. X-ray photoelectron spectra (XPS) were performed on an AXIS UltraDLD system (Shimadzu-Kratos), operating at 150 W with Al Kα radiation (1486.6 eV).

### Electrochemical Measurements of Half Cell for GCNT@ST-MNC Cathode

For fabrication of the cathode, the GCNT@ST-MNC powder was mixed with Super P carbon black and polyvinylidene fluoride binder (8:1:1 in weight) in N-methyl-2-pyrrolidone. Next, the slurry was coated onto Al foil and dried overnight at 90 °C in vacuum with the loading mass of 1.3 mg cm^−2^. The half-cell tests were performed using a CR2016 coin-type cell with Li metal as the counter and reference electrode, assembled in an Ar-filled glove box. The separator was a microporous polypropylene membrane (Celgard 2500). The electrolyte was 1 mol L^−1^ LiPF_6_ in a mixture of ethylene carbonate/diethyl carbonate (1:1, V/V). Galvanostatic charge/discharge, rate performance and cycling stability were recorded on a battery test instrument (LAND CT2001A) in voltage range of 2.5–4.8 V. Cyclic voltammetry (CV) and electrochemical impedance spectroscopy (EIS) were carried out with an electrochemical workstation (CHI660E). Here, 1 C current corresponds to 377 mA g^−1^ based on the cathode mass.

### Electrochemical Measurements of Full Cell for GCNT@ST-MNC Cathode and Graphite Anode

The fabrication of nanographite anode was the same as that of the cathode, and the anode was pre-lithiated prior to use. The detailed process is described as follows: nanographite electrode was assembled with Li counter electrode in coin cell in order to make it lithiated in 1 M LiPF_6_ electrolyte. The cell was charged from the open circuit potential to 0.01 V (vs. Li/Li^+^) and then discharged to 1.0 V to complete one cycle. For nanographite lithiation, the cell was again charged to 0.01 V (vs. Li/Li^+^). After completing lithiation, the nanographite electrode was recovered by disassembling the coin cell. The GCNT@ST-MNC cathode and pre-lithiated nanographite anode were assembled in CR2016 coin cell with an N/P ratio of 1.2. The full cell was cathode limited (1 C current corresponding to 377 mA g^−1^ based on cathode mass) and galvanostatically charged/discharged in voltage range of 2.5–4.8 V vs. Li/Li^+^.

## Results and Discussion

### Morphology and Structure Characterization

**Fig. 2:**
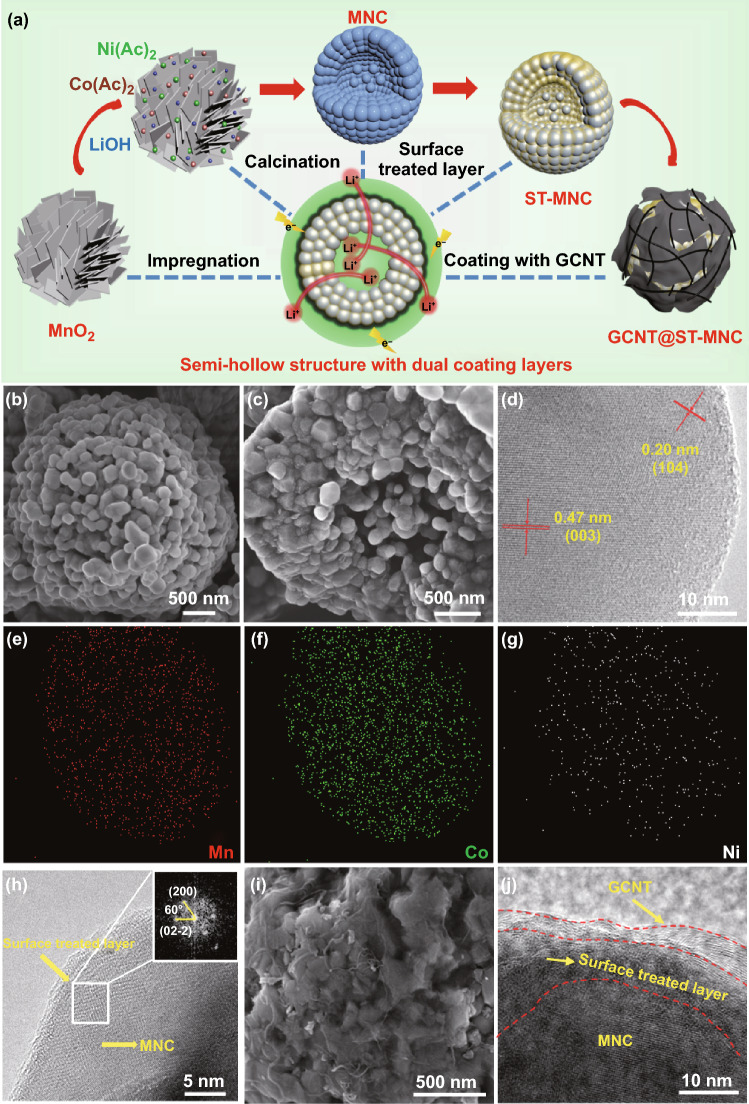
**a** Schematic illustration of preparation process of GCNT@ST-MNC composites. **b** FE-SEM and **c** FE-SEM cross-sectional images of MNC. **d** High-resolution TEM (HR-TEM) image of MNC. **e–g** EDS mapping of ST-MNC. **h** HR-TEM image of ST-MNC. The inset is the fast Fourier transformed (FFT) pattern. **i** FE-SEM and **j** HR-TEM images of GCNT@ST-MNC

Figure 2a illustrates the preparation procedure of GCNT@ST-MNC composites. Uniform MnO_2_ microspheres composed of stacked nanosheets are firstly prepared by thermal decomposition of MnCO_3_ at 400 °C. Owing to the release of CO_2_, the obtained MnO_2_ microspheres are highly porous (Fig. S2a, b). Next, semi-hollow MNC microspheres are obtained by impregnating Co(Ac)_2_, Ni(Ac)_2_ and LiOH into MnO_2_ and solid-state calcination. The obtained MNC microspheres consist of nanosized primary particles of ca. 200 nm (Fig. 2b), in which hollow holes are observed around the loosely connected primary particles in the core, due to the interplay between fast outward diffusion of metal atoms and slow inward diffusion of O atom (Fig. 2c) [34]. To obtain a statistical result of the morphology the MNC microspheres, 9 arbitrary SEM images further showed similar semi-hollow structures (Fig. S2c-k). HR-TEM images clearly reveal the lattice fringes with interplanar spacings of 0.47 and 0.20 nm, which correspond to the (003) and (104) planes of MNC nanocrystals, respectively (Fig. 2d). To ensure better interphase stability and improve the Li^+^ diffusion, the surface of the MNC sample is firstly modified by a simple reported ionized solution coating method (ST-MNC) [40]. We chose Co-containing coating layers for the surface treatment considering that they contribute to improve the electrochemical performance of layered cathodes [26, 40]. It has been reported that the using the completely ionized solution with lithium and cobalt acetate in ethanol to conduct the surface treatment could result in a stable Li_x_CoO_2_ phase after heating treatment [40]. Before coating, the surface of the pristine MNC material (Fig. S3) is smooth and only shows the lattice fringes of the MNC crystal, no other species were observed at the surface. By contrast, a thin layer with different lattice fringes was found to adhere to the outer surface of MNC primary particles (Fig. 2h), indicating formation of the surface treated layer (ST-MNC). The fast Fourier transformed (FFT) pattern further confirmed the phase of the surface-treated layer belonged to the space group of Fm-3 m, which is identical to the reported result [40]. EDS mapping analysis along with inductively coupled plasma (ICP) measurement (Figs. 2e-g, S4 and Table S1) further shows that a higher Co content in the surface coating layer. To achieve electron conductive GCNT coating, GOCNT dispersion was dropwise added into ST-MNC and then hydrazine reduction and annealing were carried out. As seen from Fig. 2i, graphene nanosheets are tightly covered on ST-MNC surface with uniformly distributed CNTs for GCNT@ST-MNC composite. The long high aspect ratio of CNT helps to build long-range electron conductive network to boost the rate performance. The above method successfully acquires a thin GCNT coating on ST-MNC surface with a thickness of 3–4 nm (Fig. 2j), which construct fast IEPs in the composite. TGA result determines the GCNT content in GCNT@ST-MNC to be ca. 4% (Fig. S5). Importantly, it is found that GCNT@ST-MNC reveals a tap density as high as 2.1 g cm^−3^. Compared with completely hollow microsphere with large cavities inside the core, the semi-hollow microsphere is assembled from the close stacked outer shell and loosely connected particle occupied the inner core. Therefore, the utilization of the space per unit volume is improved.

**Fig. 3:**
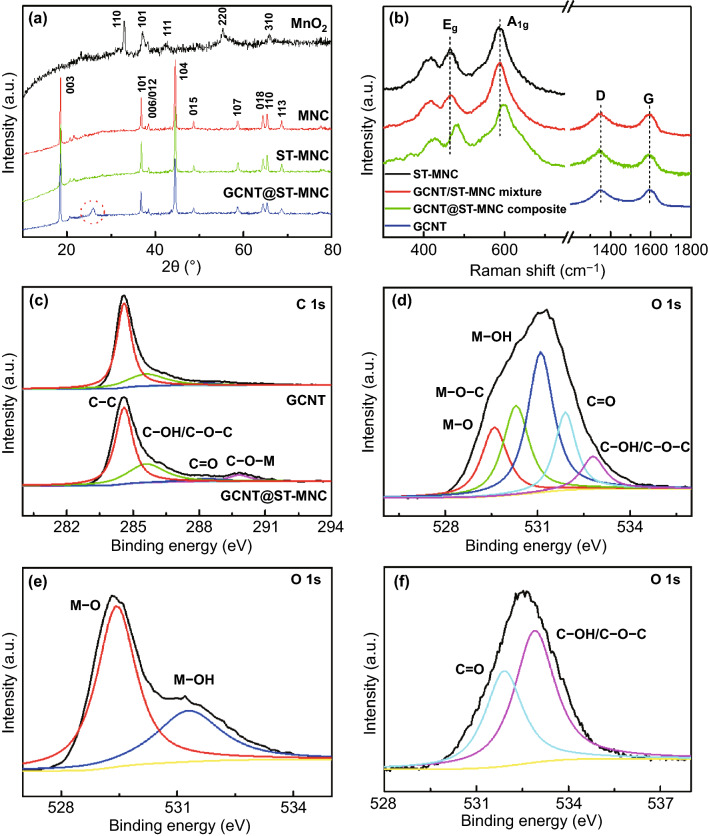
**a** XRD patterns. **b** Raman spectra. **c** High-resolution C 1 s XPS spectra of GCNT and GCNT@ST-MNC. High-resolution O 1 s XPS spectra of **d** GCNT@ST-MNC, **e** ST-MNC and **f** GCNT

The crystal/phase structure of the materials was characterized by XRD (Fig. 3a). The obtained MnO_2_ reveals a pure tetragonal phase (JCPDS No. 01–0799). After calcination, the MNC shows the typical pattern of hexagonal α-NaFeO_2_ structure with the R-3 m space group [41]. The clear split in 006/012 and 018/110 pair peaks also indicates superior crystallinity and good hexagonal ordering [42]. The weak reflection peaks indexed with C2/m space group appear at 2θ = 20° ~ 25°, which originates from the ordering of metal ions in layered lattice of LMR oxides [43]. After surface treatment, the crystal structure keeps almost unchanged for ST-MNC. Besides, an extra weak peak at 26° for GCNT@ST-MNC is assigned to the graphite-like structure, which reflects the presence of GCNT coating on ST-MNC surface. The main XRD peaks are enlarged in Fig. S6a to examine the influence of modification on crystal structure of the MNC material. It was observed that XRD peak positions remained almost unchanged, indicating that the bulk structure of the modified MNC was not affected. X-ray Rietveld refinement (Fig. S6b-d) of the MNC, ST-MNC and GCNT@ST-MNC was performed to further compare the variation of lattice parameters. The detailed structural parameters are summarized in Fig. S7. The changes in lattice parameters do not exceed 0.05% for both ST-MNC and GCNT@ST-MNC, implying that the surface modification does not alter the bulk structure and the surface-treated layer just exists on the MNC surface, rather than incorporates into the MNC host. Besides, the *c/a* ratios of MNC, ST-MNC and GCNT@ST-MNC materials all are ~ 4.99, higher than the critical value of 4.89, indicating that they have good layered structures. It should be noted that the intensity ratio of *I* (003)/*I*(104) for the GCNT@ST-MNC is higher than that of the MNC, indicating that the dual coating process is favourable for reducing the degree of cation mixing. This might be attributed to the fact that loner calcination time of GCNT@ST-MNC than the MNC could result in better crystallinity with well-defined layered characteristics and less cation mixing degree [41]. In addition, hydrazine treatment during the GCNT coating has been found to effectively stabilize the surface of the Li_2_MnO_3_ phase and suppress TM ion migration to the Li slab [7, 19], which is also beneficial to reduce the degree of cation mixing. Raman spectra were also used to distinguish the conformal coating layer induced structural change. The prominent peak of MnO_2_ at 633 cm^−1^ is attributed to the symmetric Mn–O lattice vibration (Fig. S8). The MNC displays two classical Raman-active *A*_1g_ and *E*_g_ peaks of LMR oxide at 590 and 475 cm^−1^, which is assigned to symmetrical stretching and deformation of M–O, respectively [44]. Moreover, the non-splitted *A*_1g_ peak indicates good integration of Li_2_MnO_3_ and LiMO_2_ region for MNC. After GCNT coating, *A*_1g_ and *E*_g_ peaks shift to higher wavenumbers, with increments of 20 and 16 cm^−1^, respectively, which could be induced by doping effect and/or bonding formation in GCNT@ST-MNC composite (Fig. 3b). Given that for physically mixed composites, the peak positions of *A*_1g_ and *E*_g_ are usually unchanged [45], and it is speculated that the Raman shifts observed in GCNT@ST-MNC would indicate the formation of C-O-M linkage. Moreover, a larger *I*_D_/*I*_G_ ratio also indicates the increase in disorder degree and reflects to some extent the adhesion and interaction between GCNT and ST-MNC. Further evidence can also be observed in FTIR spectra (Fig. S9).

To evaluate the effect of surface coating on the TM’s (Ni, Co, Mn) valence state, XPS analysis was conducted to detect and compare the valence state of the TMs in the NMC, ST-NMC and GCNT@ST-MNC materials (the detail sees in Fig. S10). All the three samples exhibited nearly identical peak positions in the XPS spectrum, implying that the small amount of (~ 4 wt%) GCNT coating has no impact on the valence state of the TMs. It's worth noting that compared with MNC, Co 2p signal of ST-MNC obviously strengthens, which is due to the more amount of Co on the surface (Fig. S11), consistent with the ICP and EDS results. Gaussian fit to C 1 s spectrum of GCNT shows three peaks, C–C/C = C (284.6 eV), C–OH/C–O–C (285.6 eV) and C = O (288.3 eV) [45–47], respectively (Fig. 3c). In contrast, an extra peak appears at 289.8 eV for GCNT@ST-MNC, which is ascribed to the C-O-M bond [45]. The fitting of O 1 s spectra shows that the peaks of GCNT@ST-MNC corresponding to M–O/M-OH groups (529.6/531.1 eV) are consistent with those of ST-MNC (Figs. 3d-e). The peaks at 531.9 and 532.8 eV are attributed to C = O and C–OH/C–O–C groups, respectively (Fig. 3f) [45, 46]. Compared with GCNT, the peak intensity of C–OH/C–O–C groups of GCNT@ST-MNC significantly becomes small, suggesting the replacement of H atom in -OH group or ring-opening of C–O–C group to form C-O-M linkage [46, 47]. Coincidentally, a new peak also appears at 530.3 eV and may be attributed to the C-O-M bond. Moreover, due to the absence of C-M bond in C 1 s or metal 2p spectra, oxygen-containing groups can be regarded as a medium to anchor GCNT on ST-MNC through the C-O-M linkage. Apparently, such chemical-bonded GCNT on ST-MNC is beneficial to stabilize the whole particle, which reduces the possibility of microcrack generation. Moreover, the corrosion resistance endowed by graphene can largely avoid side reactions with electrolyte or acid.

### Electrochemical Performance Enhancement Enabled by the Bifunctional Strategy

**Fig. 4:**
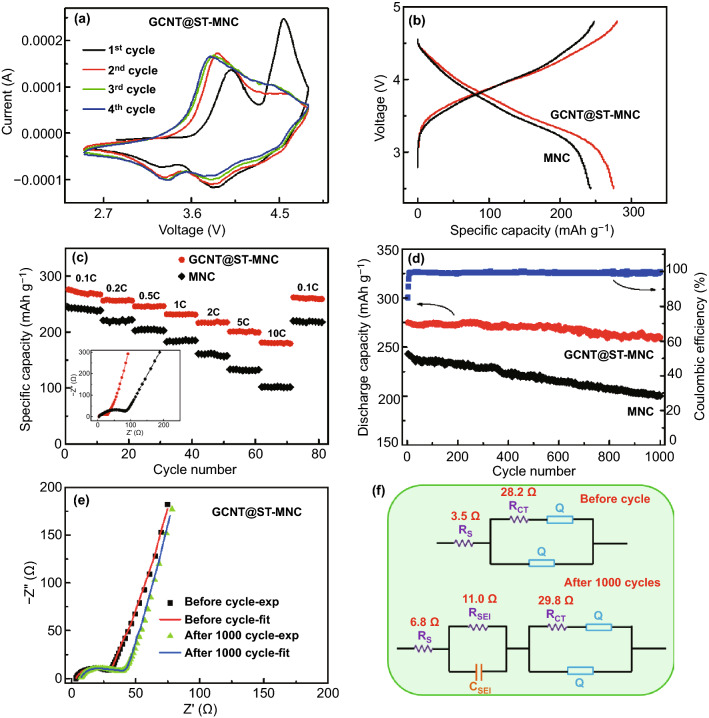
Electrochemical performance of MNC and GCNT@ST-MNC half cells. **a** CV curves of GCNT@ST-MNC from 1st and 4th cycle at 0.1 mV s^−1^. **b** Galvanostatic charge/discharge curves at 0.1 C. **c** Rate performance at various rates from 0.1 to 10 C (the inset is EIS measurement). **d** Cycling performance at 0.1 C (Coulombic efficiency of GCNT@ST-MNC). **e** EIS of GCNT@ST-MNC before cycle and after 1000 cycles with experimental and fitting data. **f** Equivalent circuits for EIS fitting of GCNT@ST-MNC before cycle and after 1000 cycles

The unique morphology and dual coatings of GCNT@ST-MNC are beneficial for constructing conformal particle for both enhanced interphase and structure integrity to improve the electrochemical performance. As seen from CV curves of GCNT@ST-MNC, Li_2_MnO_3_ was fully activated during the 1st cycle as charged to above 4.5 V and the corresponding anodic peak appears at ca. 4.5 V, which is consistent with the obvious charging plateau (Figs. 4a and S12) [27] Another anodic peak at 4.0 V is attributed to Ni^2+^ and Co^3+^ being oxidized to tetravalent states. Accordingly, two cathodic peaks appear at 3.3 and 3.8 V due to the redox reactions of metal ions. For the 2^nd^ cycle, however, the CV peak and charging plateau at 4.5 V almost disappear, and the activation process provides a discharge capacity up to 275 mAh g^−1^ at 0.1 C (Fig. 4b), which is much higher than traditional LiMO_2_ and most LMR oxides (Table S2) [19, 21, 23, 27, 48–52]. With the activation of the MNC, the subsequent CV curves exhibit smaller voltage gap between the two cathodic peaks located at ~ 3.3 and ~ 3.8 V, implying less polarization and favourable reaction kinetics enabled by the dual coating layers. The initial CE of the GCNT@ST-MNC is 87.8%, while for the uncoated MNC, the CE is only 77.6%. The significant enhancement of the initial CE indicated that the dual surface coatings were helpful to activate the MNC (Fig. S12a, b). Moreover, its high tap density (GCNT@ST-MNC, 2.1 g cm^−3^) brings about an ultrahigh volumetric energy density (based on cathode) of 2234 Wh L^−1^, which is apparently related to the unique semi-hollow structure of MNC microspheres and the activation of Li_2_MnO_3_. Rate performance of half cell was measured at different applied currents (Fig. 4c). The specific capacity at different rates was converted the to the normalized capacity by that at 0.1 C for convenience to compare the rate capability of the two samples (Fig. S12c). The specific capacity retention of the GCNT@ST-MNC at each rate is higher than that of MNC. For example, GCNT@ST-MNC retains 65.5% capacity (180 mAh g^−1^) at 10 C, while MNC only holds 41.6%; meanwhile, a highly reversible capacity (261 mAh g^−1^) was restored as re-discharged at 0.1 C. This result indicates that the conformal dual layer coating with good IEPs is highly important to boost the Li deintercalation/intercalation kinetics, compared with single morphology-strategy modulated MNC. Electrochemical impedance spectroscopy (EIS) measurement further confirms that GCNT@ST-MNC displays a relatively small semicircle in the high-frequency region, suggesting low charge-transfer resistance (*R*_CT_) and good electrical conductivity (the inset of Fig. 4c).

In addition, GCNT@ST-MNC reveals excellent cycling stability, with 94.5% retention after 1000 cycles (only 0.055‰ decay per cycle) and nearly 100% Coulombic efficiency, in sharp contrast with 17.6% capacity loss of MNC (Fig. 4d). Such cycling performance is far superior to most reported results, especially given that those reported results were mostly measured by 100 cycles (Table S2). To further verify conformal structure endowed excellent cycle performance, EIS before and after cycle was measured, for which equivalent circuit was used for fitting (Fig. 4e, f) [21]. It is apparent that GCNT@ST-MNC only exhibits small circular arc (*R*_CT_) and nearly unchanged straight line slope after 1000 cycles. By fitting, the ohmic resistance (*R*_S_) is determined as 3.5 Ω before cycle, which increases to 6.8 Ω after 1000 cycles. The *R*_CT_ exhibits a nearly identical value during cycling. Also, the generated solid-electrolyte interface resistance (*R*_SEI_) is merely 11 Ω. More importantly, the morphology GCNT@ST-MNC reveals hardly discernable change even after 1000 cycles (Fig. S13). Furthermore, we evaluated the stability of the dual surface coatings after 200 and 500 cycles at 0.1 C by HRTEM (Fig. S14). The results show that the GCNT layer and the surface-treated layer were well maintained and tightly adhered to the surface of MNC after long cycling. Besides, the CV curves (Fig. 16a) of GCNT@ST-MNC showed distinct redox reaction peaks even after 1000 cycles. This implies that the dual surface coating layers played excellent protection role and retarded side reactions during cycling. The electrochemical performance (including rate, cycle performance and charge/discharge profiles) of ST-NMC was shown in Fig. S15a-c. Although the sample has been treated by surface layer, the ST-MNC still exhibited lower rate and cycle performance than the GCNT@ST-MNC. This result further indicates the such dual coating layers are effective for suppressing structure collapse and boosting fast electron transfer, and thus contribute to achieve high-performance MNC. The above results unambiguously indicated that the effectiveness of the bifunctional strategy in constructing high-conformal structure and suppressing side reactions with electrolyte.

**Fig. 5:**
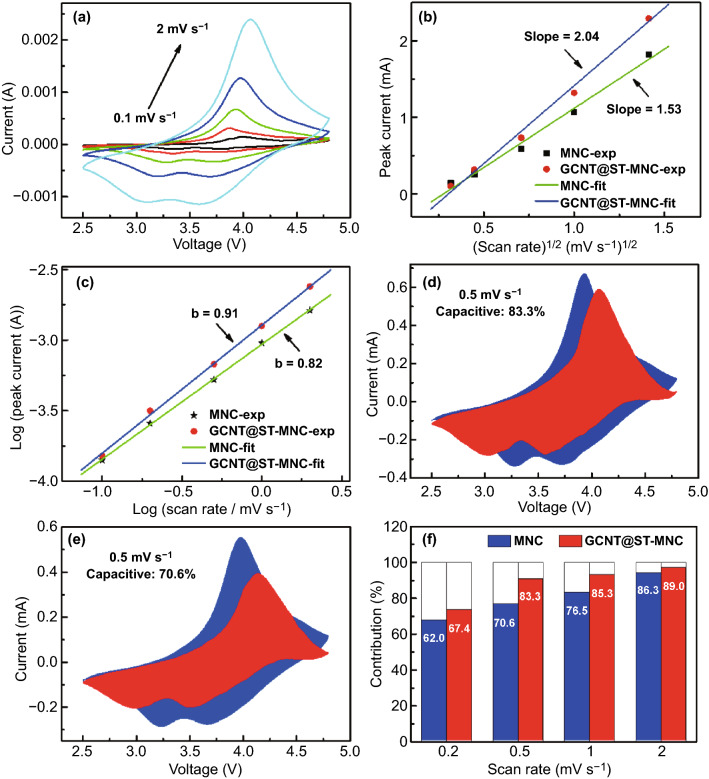
**a** CV curves of GCNT@ST-MNC at various scan rates from 0.1 to 2 mV s^−1^. **b** Plotting of peak current (*I*_p_) vs. square root of scan rate (*v*^1/2^). **c** Plotting of log(peak current, *I*_p_) vs. log(scan rate, *v*) (*b* refers to the slope of fitting line). Total current (blue region area) and capacitive current (red region area) at 0.5 mV s^−1^ for **d** GCNT@ST-MNC and **e** MNC. **f** Comparison of capacitive contributions at various scan rates

To further understand the Li^+^ diffusion behavior, Li^+^ diffusion coefficient (*D*_Li+_) and reaction kinetics were studied by CV scanning at rates from 0.1 to 2 mV s^−1^ (Figs. 5a and S15d). The CV curve comparison (Fig. S16) of GCNT@MNC and MNC was firstly conducted. The GCNT@MNC exhibited smaller voltage gap between the two cathodic peaks and larger peak current values relative to the MNC at different scan rates (0.1 and 1 mV s^−1^). This reflects the better reaction kinetics in GCNT@MNC, and further indicates that the dual coatings are beneficial to improve the electrochemical performance. Obviously, the voltage difference between redox peaks gradually enlarges with the increase in scan rate, indicating the ever-increasing polarization. The relationship between peak current (*I*_p_) and scan rate (*v*) can be described by Randles–Sevcik equation: *I*_p_ = 2.69 × 10^5^*n*^3/2^*AD*^1/2^*v*^1/2^*C* (the details are included in Supporting information) [53]. As shown in Fig. 5b, *I*_p_ is nearly proportional to *v*^1/2^ for both MNC and GCNT@ST-MNC, indicating that the electrode process is rate-determined by Li^+^ ion diffusion [54]. Given that *n*, *A,* and *C* are constant, the slope of *I*_p_ vs. *v*^1/2^ is directly related to *D*_*Li*+_. As a result, compared with 1.53 for MNC, a higher slope value (2.04) for GCNT@ST-MNC implies more efficient Li^+^ diffusion, agreeing with excellent rate capability. In addition, Li^+^ intercalation kinetics can be analyzed by plotting log*I*_p_ vs. log*v* (obeying the relationship with *I*_p_ = *a*·*v*^*b*^), where *b* = 0.5 or 1.0, implying a semi-infinite diffusion or capacitive process, respectively [55]. For GCNT@ST-MNC, *b* is found to be 0.91 (close to 1) and higher than 0.82 of MNC, suggesting a faster Li^+^ insertion process with a typical capacitive behavior, (Fig. 5c). Such capacitive kinetics can be further evaluated by *i(V)* = *k*_*1*_*v* + *k*_*2*_*v*^1/2^, see Supporting information. The surface capacitance (*k*_*1*_*v*) is marked as red region in the whole blue CV area. Apparently, the capacitance contribution ratio (83.3%) for GCNT@ST-MNC is higher than 70.6% for MNC at 0.5 mV s^−1^ (Fig. 5d, e). The ratio gradually increases with increasing scan rates and reaches 89.0% at 2 mV s^−1^, implying that the capacitive storage for GCNT@ST-MNC plays a dominant role at each scan rate (Fig. 5f). These results indicate that the dominant capacitive storage mechanism endows GCNT@ST-MNC cathode with outstanding reaction kinetics [55].

Based on the above analysis, we consider the excellent performance of GCNT@ST-MNC arising from the bifunctional strategy modulation. From the structure point of view: (1) the primary nanoparticle shortens the Li diffusion paths; (2) the abundant space in the inner core provides large accessible surface; (3) the semi-hollow structure has good self-adaption capability to relieve the volumetric change. From the surface modification point of view: (1) the GCNT/cation-mixing dual protective layers provide fast IEPs; (2) the high mechanical property and good corrosion resistance of GCNT as the outer layer suppress side reactions with electrolyte; (3) the GCNT covalently anchored on ST-MNC mitigates the detachment of MNC and diminishes the occurrence of microcracks by suppressing structural change. These constitute a synergetic effect that enables GCNT@ST-MNC cathode to operate at higher rates and exhibit higher gravimetric/volumetric capacities and cycling stability, which can be further highlighted by the performance comparison with other reported LMR cathodes (Table S2).

### Full Cell Assembly and Energy Density Assessment

**Fig. 6:**
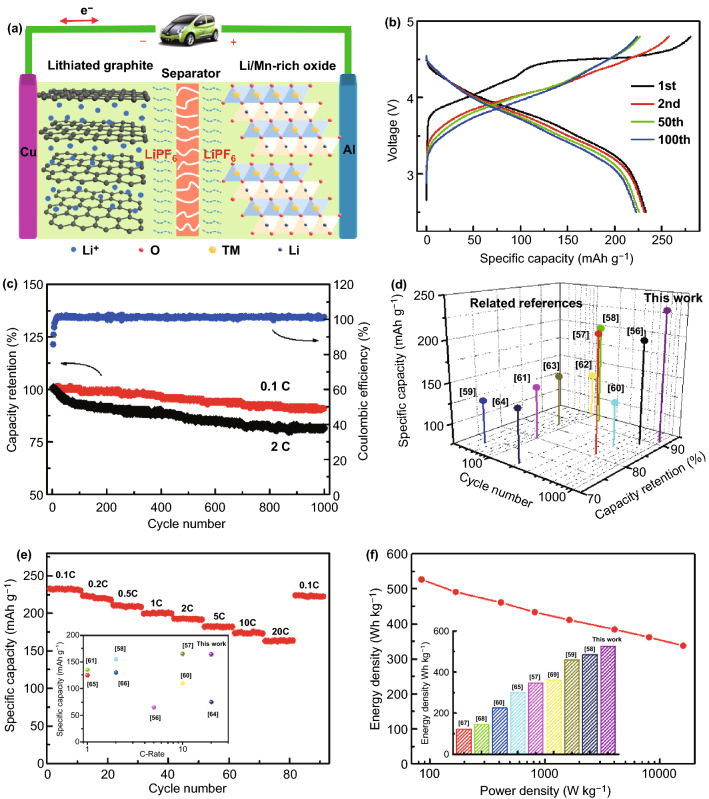
Electrochemical performance of GCNT@ST-MNC//nanographite full cell. **a** Schematic of the assembled full cell. **b** Galvanostatic charge/discharge curves with 1st, 2nd, 50th and 100th cycle at 0.1 C. **c** Cycling performance at 0.1 C and 2 C (Coulombic efficiency at 0.1 C). **d** Comparison of specific capacity and cycling performance of our work with other reported full cells. **e** Rate performance at various rates from 0.1 to 20 C (the inset is the comparison of specific capacity at high rate). **f** Ragone plots based on total mass of cathode and anode (the inset is the comparison of energy density)

To further demonstrate the superiority of GCNT@ST-MNC, we assembled full cells with nanographite anode with a low negative/positive (N/P) ratio of 1.2. The mass loading of the cathode is ~ 5 mg cm^−2^. The schematic is shown in Fig. 6a. Based on the cathode mass, the full cell displays a discharge capacity of 233 mAh g^−1^ at 0.1 C for 1st cycle and only 4% capacity loss after 100 cycles (Fig. 6b). For prolonged cycles, it achieves 91% and 82% capacity retention at 0.1 C and 2 C after 1000 cycles, respectively (Fig. 6c). The excellent electrochemical performance also reflects on almost unchanged EIS curves before and after cycling (Fig. S17). For comparison, the results of the reported full cells are also included in Fig. 6d. It can be seen that most of the reported full cells exhibit low capacity retentions even at shorter cycles [56–64]. As the current increases to 20 C, as shown in Fig. 6e, the capacity of GCNT@ST-MNC cell can still reach 164 mAh g^−1^, corresponding to a 70.3% retention; the inset displays capacity comparison of the reported results at high rate [56–58, 60, 61, 64–66].

For advanced LIBs, energy density is an important performance indicator. GCNT@ST-MNC full cell reveals an energy density up to 874 Wh kg^−1^ at the power density of 85 W kg^−1^, based on the cathode mass. When based on the total mass of cathode and anode, the energy density of the full cell is still 526.5 Wh kg^−1^, which still retains 337.6 Wh kg^−1^ even at an ultrahigh power density (15.9 kW kg^−1^, Fig. 6f). Such high energy and power densities are highly desired for fast-charging electrical vehicles, given that the power density of GCNT@ST-MNC full cell is comparable to that of supercapacitors. Meanwhile, the energy density of GCNT@ST-MNC full cell is superior to those of reported results (see the inset of Fig. 6f) [57–60, 65, 67–69].

Consequently, several aspects merit consideration in developing high performance Li/Mn-rich-based full cells. (1) A optimized morphology combined with compatible surface modifications contribute to construct conformal structure, ensuring high-efficiency utilization of the cathode material and facilitate charge (ion and electron) transport within the whole electrode; (2) a wide operating voltage can effectively enhance the energy density of the cell through the synergy of cathode and anode. For example, nanographite anode possesses high capacity (406 mAh g^−1^) and low discharging plateau (0.08 & 0.11 V) (Fig. S18), which is beneficial for widening operating voltages (ca. 3.7 V on average); (3) an optimized *m*(anode)/*m*(cathode) ratio or N/P ratio can maximize the capacity of active materials and effectively avoid excessive charge/discharge.

## Conclusions

In summary, semi-hollow Li/Mn rich Li_1.2_Mn_0.54_Ni_0.13_Co_0.13_O_2_ (MNC) microspheres were prepared through the MnO_2_ template and modified by surface-treated layer and GCNT dual coatings. The semi-hollow structure is composed of outer closely packed shell and loosely connected nanoparticles in the inner core. Such structure design revealed enhanced tap density, mitigated volume expansion and bidirectional ion transport highways. Moreover, the dual surface coatings not only enhanced the mechanical stability of the cathode, but also suppressed side reactions with electrolyte. The resulting MNC cathode exhibited a capacitive-dominant charge-storage behavior (89% at 2 mV s^−1^) and high volumetric energy density (2234 Wh L^−1^) comparable to LiCoO_2_ cathode. When assembling into a full cell with the nanographite anode, it achieved 91% capacity retention after 1000 cycles and high energy/power densities of 526.5 Wh kg^−1^/15.9 W kg^−1^. The power density surpasses the power target for LIBs, and the energy density is also superior to the majority of reported results. It is believed that such a bifunctional strategy to design high-conformal cathodes can also be applied to other LIB electrodes to meet demands of a variety of power and energy devices.

## Supplementary Information

Below is the link to the electronic supplementary material.

Supplementary file1 (PDF 1915 kb)
