# Supplementary material for: A Bifunctional-Modulated Conformal Li/Mn-Rich Layered Cathode for Fast-Charging, High Volumetric Density and Durable Li-Ion Full Cells
Source: Nanomicro Lett. 2021 May 2;13:118. doi: 10.1007/s40820-021-00643-1 (PMC8089071; doi:10.1007/s40820-021-00643-1)
Supplement: Supplementary file 1 — Supplementary file1 (PDF 1915 kb) [file 40820_2021_643_MOESM1_ESM.pdf]

Nano-Micro Letters

Supporting Information for

A Bifunctional-Modulated Conformal Li/Mn-Rich Layered Cathode for Fast-Charging, High Volumetric Density and Durable Li-Ion Full Cells

Zedong Zhao[1, ‡], Minqiang Sun[1, ‡], Tianqi Wu[1], Jiajia Zhang[1], Peng Wang[1], Long Zhang[1], Chongyang Yang[3, *], Chengxin Peng[2, 4, *], Hongbin Lu[1, *]

[1]State Key Laboratory of Molecular Engineering of Polymers, Department of Macromolecular Science, Collaborative Innovation Center of Polymers and Polymer Composites, Fudan University, 2005 Songhu Road, Shanghai 200438, P. R. China

[2]School of Materials Science & Engineering, University of Shanghai for Science and Technology, Shanghai 200093, P. R. China

[3]National Engineering Research Center for Supercapacitor for Vehicles, Shanghai Aowei Technology Development Co., Ltd., Shanghai 201203, P. R. China

[4]Key Laboratory of Advanced Energy Materials Chemistry (Ministry of Education), College of Chemistry, Nankai University, Tianjin 300071, P. R. China

[‡]Zedong Zhao and Minqiang Sun contributed equally to this work.

*Corresponding authors. E-mail: hongbinlu@fudan.edu.cn (Hongbin Lu); cxpeng@usst.edu.cn (Chengxin Peng); ycy6142@sina.com (Chongyang Yang)

S1 Li^+ Diffusion Coefficient (D_{Li+})

The relationship between peak current (I_p) and scan rate (v) is described by Randles-Sevcik equation as follows:

$$I_p = 2.69*10^5 n^{3/2} A D^{1/2} v^{1/2} C$$

where n is the number of electrons transferred per mole of molecule, A is the active surface area per unit mass of electrode (cm^2 g^{-1}), C is the initial concentration of Li^+ in MNC crystal (mol cm^{-3}), which is deemed as the same for MNC and GCNT@ST-MNC; D is the Li^+ diffusion coefficient (cm^2 s^{-1}).

S2 Capacitance Contribution

The reaction kinetics is analyzed by CV scan at various rates from 0.2 to 2 mV s^{-1} according to the following equation:

$$i(V) = k_1 v + k_2 v^{1/2}$$

where v is scan rate, $k_1 v$ represents surface capacitance-controlled contribution, and $k_2 v^{1/2}$ denotes diffusion-controlled contribution. The above equation can be converted into:

$$i(V)/v^{1/2} = k_1 v^{1/2} + k_2$$

Thus, the value of k_1 and k_2 can be determined by plotting $i(V)/v^{1/2}$ vs. $v^{1/2}$. And k_1 and k_2 are constant at the fixed voltage.

S3 Calculation of Energy/Power Densities

The energy density (E, Wh kg^{-1}) and power density (P, W kg^{-1}) for the full cell can be calculated using the following equations:

$$E = Q \cdot V$$

$$P = 3600\ E\ /\ \mathrm{d}t$$

in which Q (mAh g^{-1}) is the specific capacity of full cell, V (V) is the operating voltage on average, and dt (s) is the discharge time.

S4 Supplementary Tables and Figures

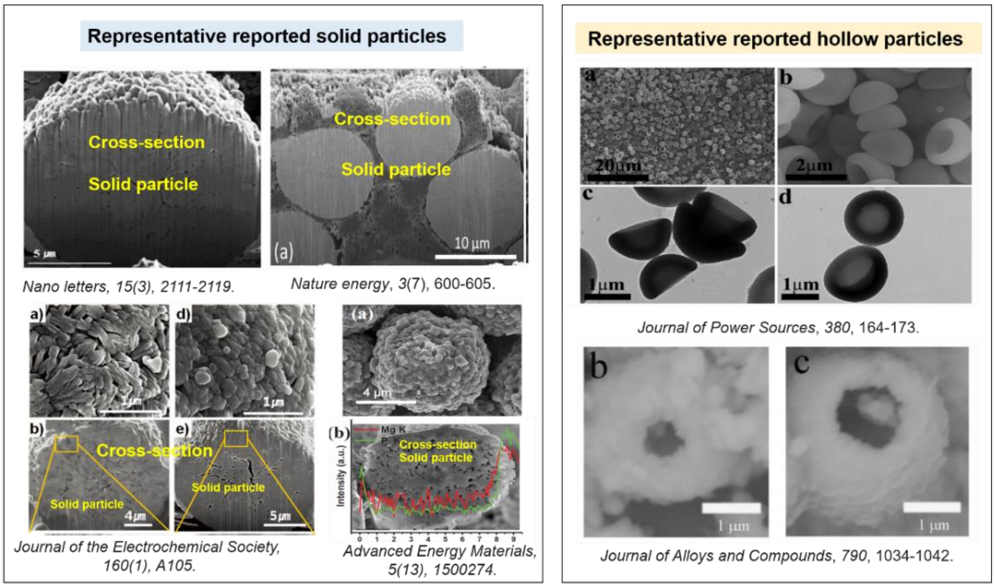

Fig. S1 Morphology and structure comparison of the solid and completely hollow-structured particles reported in references

We define the semi-hollow structure by comparing the morphology difference between the solid and completely hollow-structured particles. Figure S1(left) shows that nearly no cavities are present within the solid particles, indicating that the Li^+ diffusion is hindered obviously inside the particle, especially at high charge/discharge rates. Also, such solid particles might suffer from cracking and structure collapse during cycling, leading to fast electrode deterioration. Besides, compared with the completely hollow-structured spheres as shown in Fig. S1(right), the secondary particle packing density in the semi-hollow structure is higher in the core, thus higher tap density than the completely hollow structure. The above comparison clearly demonstrates the morphology difference among solid particles, completely hollow and semi-hollow structures.

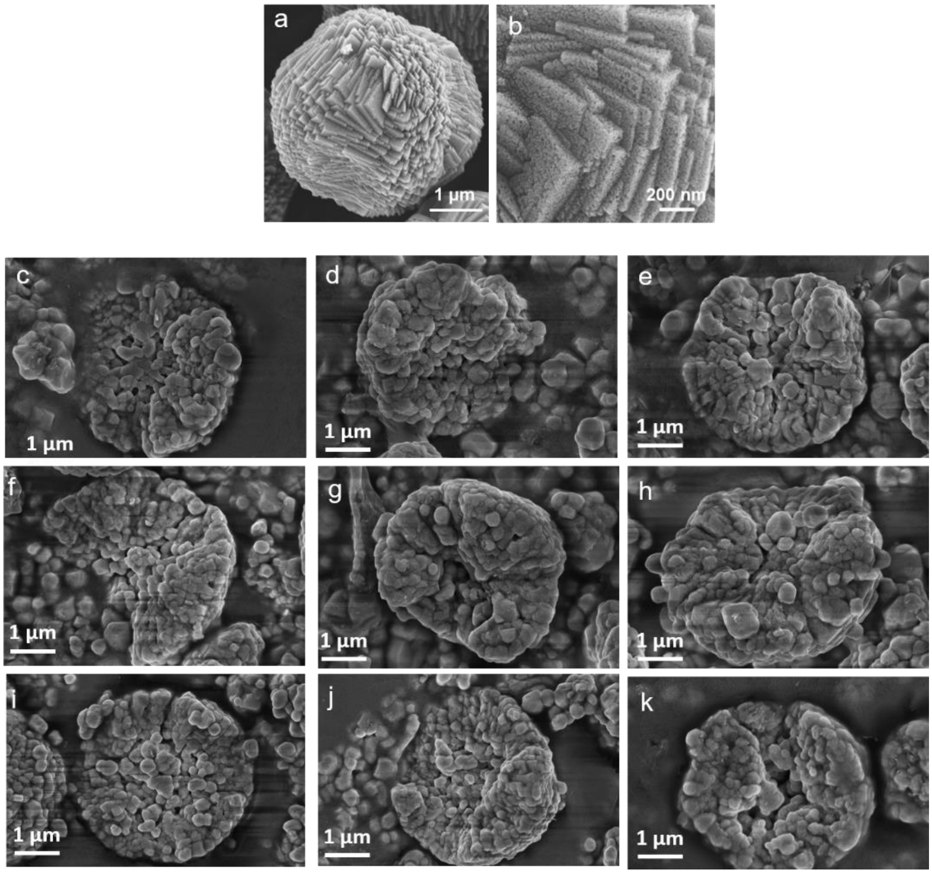

Fig. S2 FE-SEM images of MnO_2 with (**a**) low and (**b**) high magnification. (**c-k**) 9 arbitrary SEM images further showed the similar semi-hollow structures

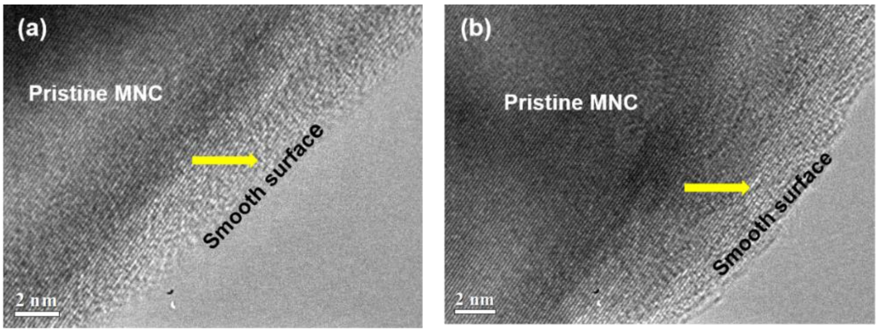

Fig. S3 (**a-b**) HRTEM images of the pristine MNC

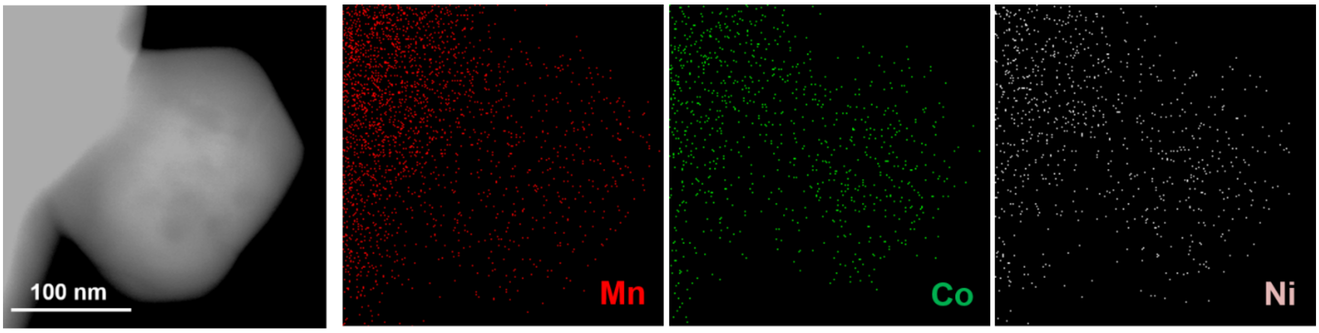

Fig. S4 EDS mapping of MNC

Table S1 Molar ratio of Li, Mn, Ni and Co for inductively coupled plasma (ICP) measurement

	Li	Mn	Ni	Co	Co: (Mn+Ni)
MNC	1.233	0.505	0.130	0.128	0.20: 1
ST-MNC	1.333	0.509	0.130	0.289	0.45: 1

EDS mapping analysis of ST-MNC reveals a relatively higher Co content across the surface region than MNC (Figs. 2e-g and S2). This is also consistent with the results of inductively coupled plasma (ICP) measurement (Table S1). The molar ratio of Li: Mn: Ni: Co is found to be 1.23: 0.51: 0.13: 0.13 for MNC, which well matches the Li1.2Mn0.54Ni0.13Co0.13O2 as expected. Moreover, the Co: (Mn+Ni) molar ratio of ST-MNC is 0.45: 1, larger than that (0.20: 1) of MNC, which suggests the Co-rich surface for ST-MNC and keeps good accordance with EDS mapping analysis.

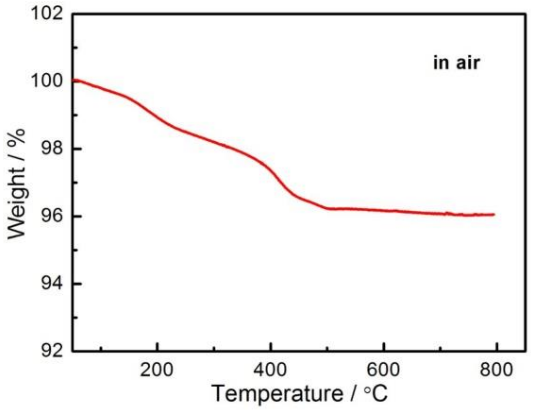

Fig. S5 TGA curve of GCNT@ST-MNC

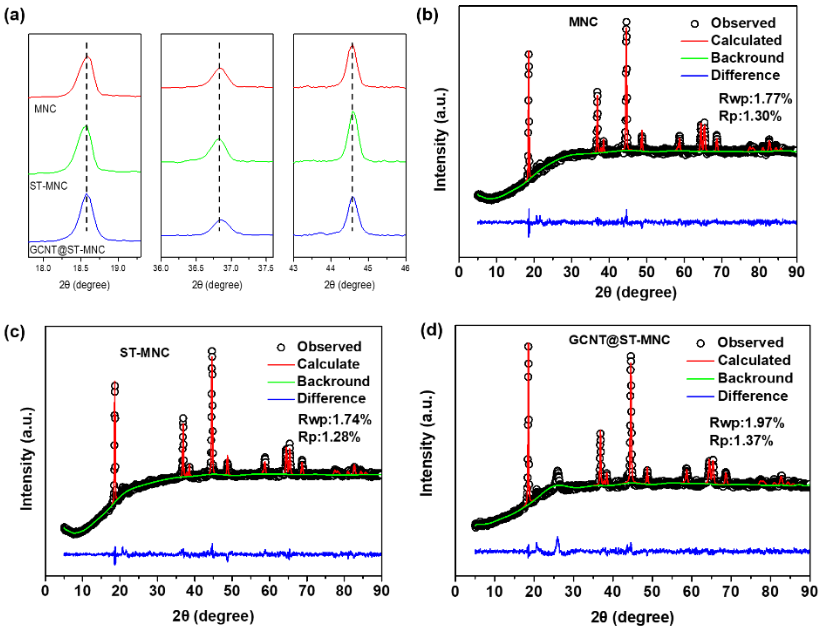

Fig. S6 (**a**) Enlarged main XRD peaks. The Rietveld refinement of XRD data of (**b**) pristine MNC, (**c**) ST-MNC and (**d**) GCNT@ST-MNC powders. The XRD analysis indicated negligible difference between these three samples.

Sample	a [Å]	c [Å]	c/a	d (003)/[Å]	d (104))/[Å]
MNC	2.8508	14.2407	4.995	4.746	2.036
ST-MNC	2.8501	14.2402	4.996	4.743	2.035
GCNT@ST-MNC	2.8502	14.2403	4.996	4.744	2.034

Fig. S7 Structural data obtained from the Rietveld refined XRD

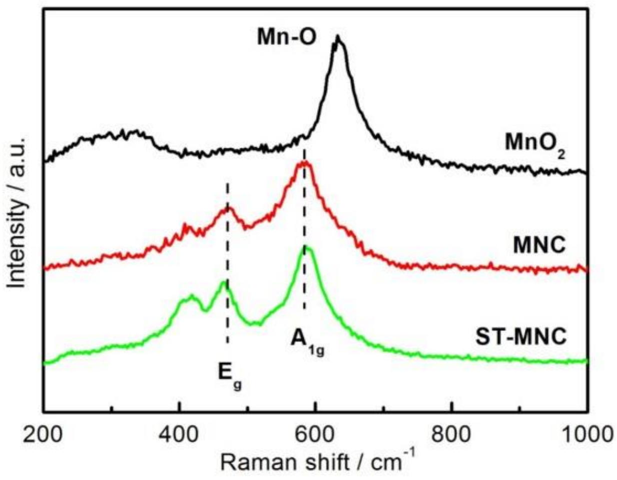

Fig. S8 Raman spectra of MnO_2, MNC and ST-MNC

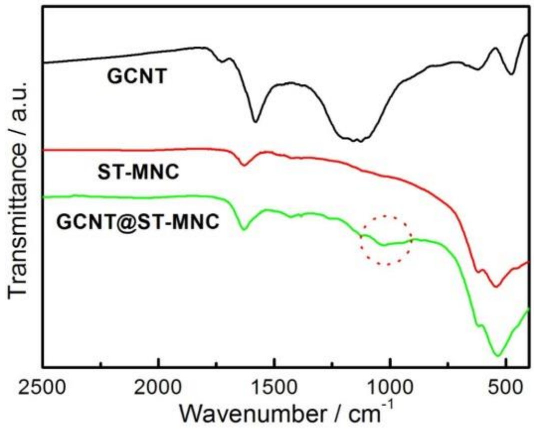

Fig. S9 FTIR spectra of GCNT, ST-MNC and GCNT@ST-MNC

In FTIR spectrum of ST-MNC, two characteristic peaks are observed at 537 and 618 cm^{-1}, which are derived from the stretching vibration of M-O. For the GCNT, the main absorption peaks include 1726 cm^{-1} (C=O in carboxyl), 1580 cm^{-1} (C=C in aromatic ring), 1200 cm^{-1} (C-O in carboxyl), 1158 cm^{-1} (C-O in C-OH) and 1092 cm^{-1} (C-O-C in epoxy group). After coating GCNT onto ST-MNC, the C=O peak at 1726 cm^{-1} and broad peaks between 1050 and 1250 cm^{-1} are absent while a new peak appears at 1027 cm^{-1} (as marked by red circle). This result verifies that the C-O-C and C-OH groups are broken down and the fresh C-O-M bond is generated between ST-MNC and GCNT.

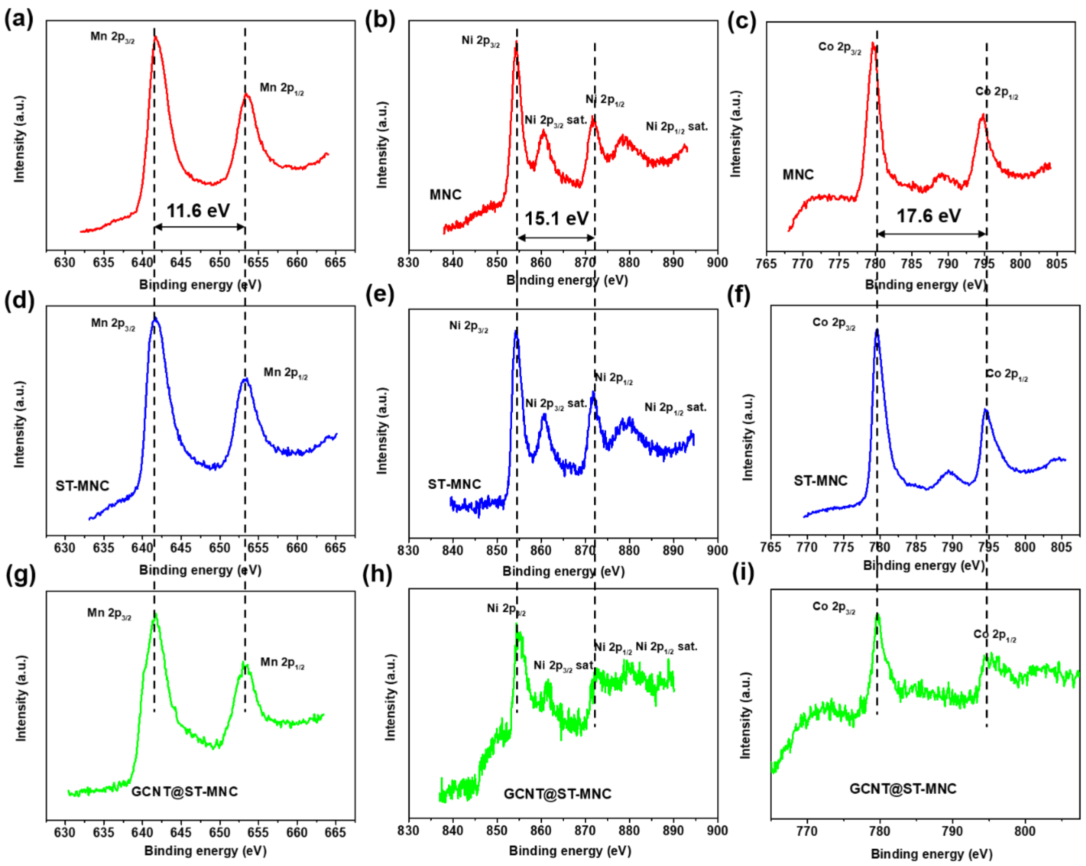

Fig. S10 XPS spectra of (**a, d** and **g**) Mn 2p, (**b, e** and **h**) Ni 2p, (**c, f** and **i**) Co 2p survey spectra for the MNC, ST-MNC and GCNT@ST-MNC

The valence state analysis of each TM is as follows. The Mn 2p spectrum (Fig. S10a, d and g) for three samples was deconvoluted into two peaks of $2p_{3/2}$ at 642 eV and $2p_{1/2}$ at 653.6 eV with a spin-energy separation of 11.6 eV, which is attributed to predominant presence of Mn^{4+} species. In the Co 2p spectrum (Fig. S10b, e and h), the binding energies of $2p_{3/2}$ and $2p_{1/2}$ peaks are 779.3 and 794.4 eV with the separation of 15.1 eV, respectively, indicating Co^{3+} valence state in the three samples. Similarly, the energy separation in Ni 2p spectrum (Fig. S10c, f and i) is 17.6 eV, corresponding to Ni^{2+} valence state. No obvious change in oxidation state for Mn, Ni and Co is identified in these three samples.

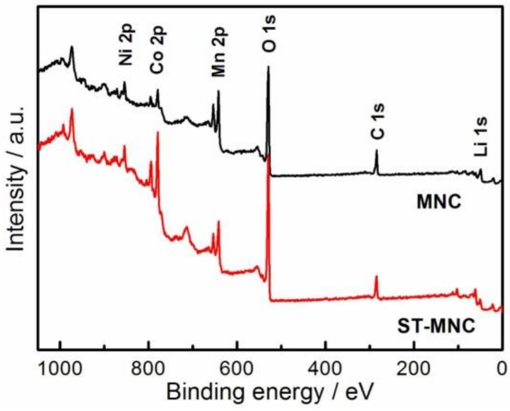

Fig. S11 Survey XPS spectra of MNC and ST-MNC

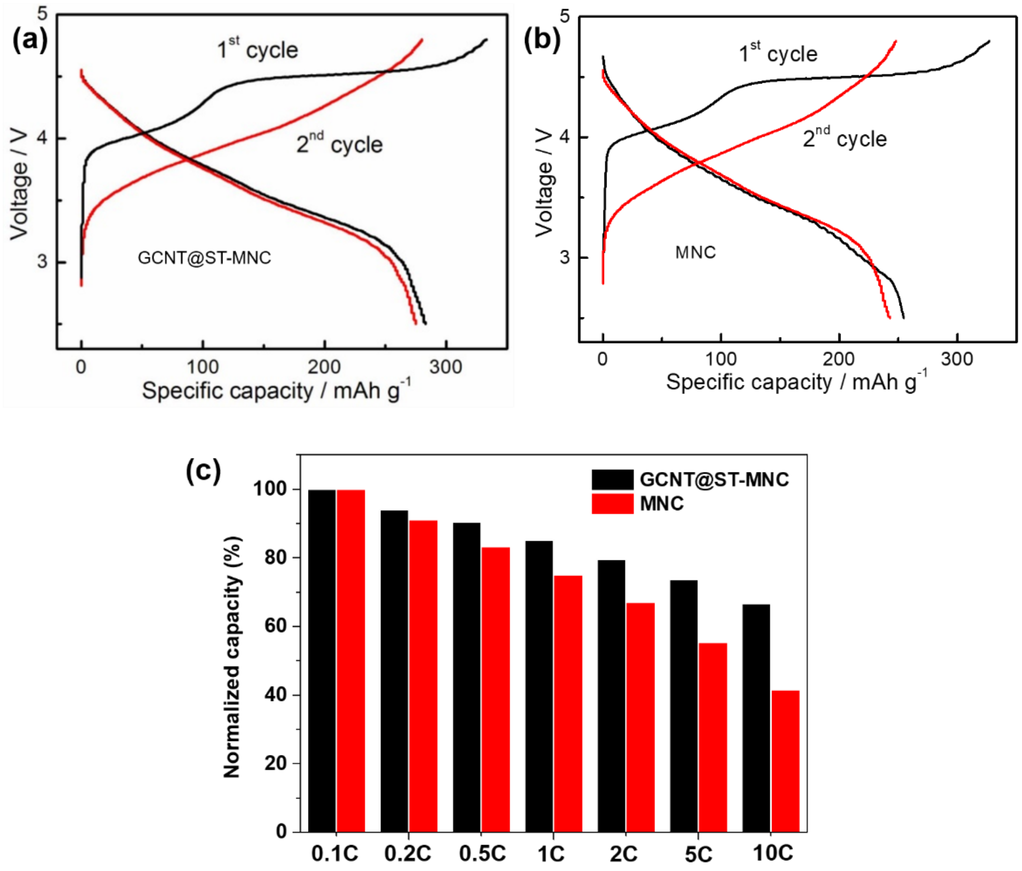

Fig. S12 (**a-b**) Galvanostatic charge/discharge curves of GCNT@ST-MNC with 1st and 2nd cycle at 0.1 C. (**c**) Normalized capacity of GCNT@ST-MNC and MNC

Table S2 Comparison of the electrochemical performance of half cells based on GCNT@ST-MNC and other recently reported $LiMO_2$ and LMR oxides

Cathode materials	Specific capacity (mAh g^{-1})	Rate capability	Cycle performance	Refs.
**GCNT@ST-MNC**	**275 (0.1 C, 2.5-4.8 V)**	**65.5% (10 C)**	**94.5% (1000 cycles)**	**This work**
Surface treated $LiNi_{0.6}Co_{0.2}Mn_{0.2}O_2$	200 (1 C, 2.75-4.4 V)	/	85% (150 cycles)	[51]
$LiNi_{0.33}Mn_{0.33}Co_{0.33}O_2$	205 (0.1 C, 2.7-4.7 V)	/	68.3% (100 cycles)	[52]
$LiNi_{0.4}Mn_{0.4}Co_{0.2}O_2$ - SWNT	160 (0.5 C, 2.5-4.5 V)	71.8% (5 C)	92% (500 cycles)	[48]
Al_2O_3 coated $LiNi_{0.4}Mn_{0.4}Co_{0.2}O_2$-CNT	160 (0.5 C, 2.5-4.5 V)	62.5% (5 C)	93% (500 cycles)	[21]
rGO caoted $0.4Li_2MnO_3$•$0.6LiNi_{0.33}Co_{0.33}Mn_{0.33}O_2$	250 (0.1 C, 2-4.6 V)	60% (12 C)	94.6% (100 cycles)	[19]

AlF_3-coated $Li_{1.2}Ni_{0.15}Co_{0.1}Mn_{0.55}O_2$	250 (0.1 C, 2-4.7 V)	/	~100% (100 cycles)	[23]
AlF_3-coated $Li_{1.19}Ni_{0.16}Co_{0.08}Mn_{0.57}O_2$	245 (0.08 C, 2-4.6 V)	56% (5 C)	91.6% (100 cycles)	[27]
TiO_2-coated $Li_{1.2}Ni_{0.13}Mn_{0.54}Co_{0.13}O_2$	310 (55 ℃) (0.1 C, 2-4.8 V)	/	70% (55 ℃) (50 cycles)	[49]
$Li_{1.2}Ni_{0.15}Co_{0.1}Mn_{0.55}O_2$	252 (21 mA g^{-1}, 2-4.8 V)	47.6% (460 mA g^{-1})	98.4% (75 cycles)	[50]

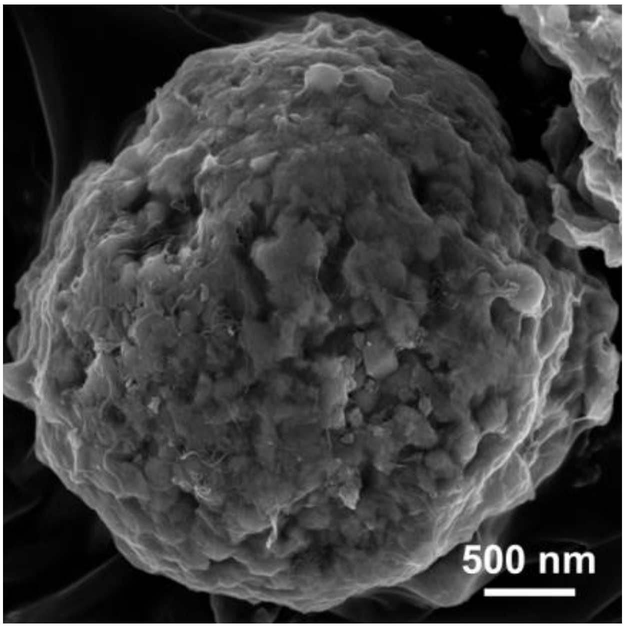

Fig. S13 FE-SEM image of GCNT@ST-MNC after 1000 cycles

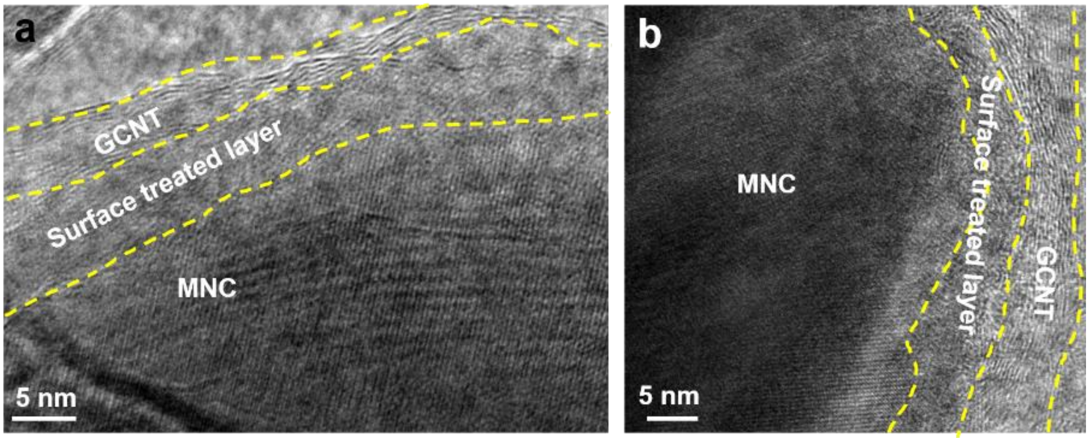

Fig. S14 The HRTEM images of the GCNT@ST-MNC after 200 (**a**) and 500 (**b**) cycles at 0.1C

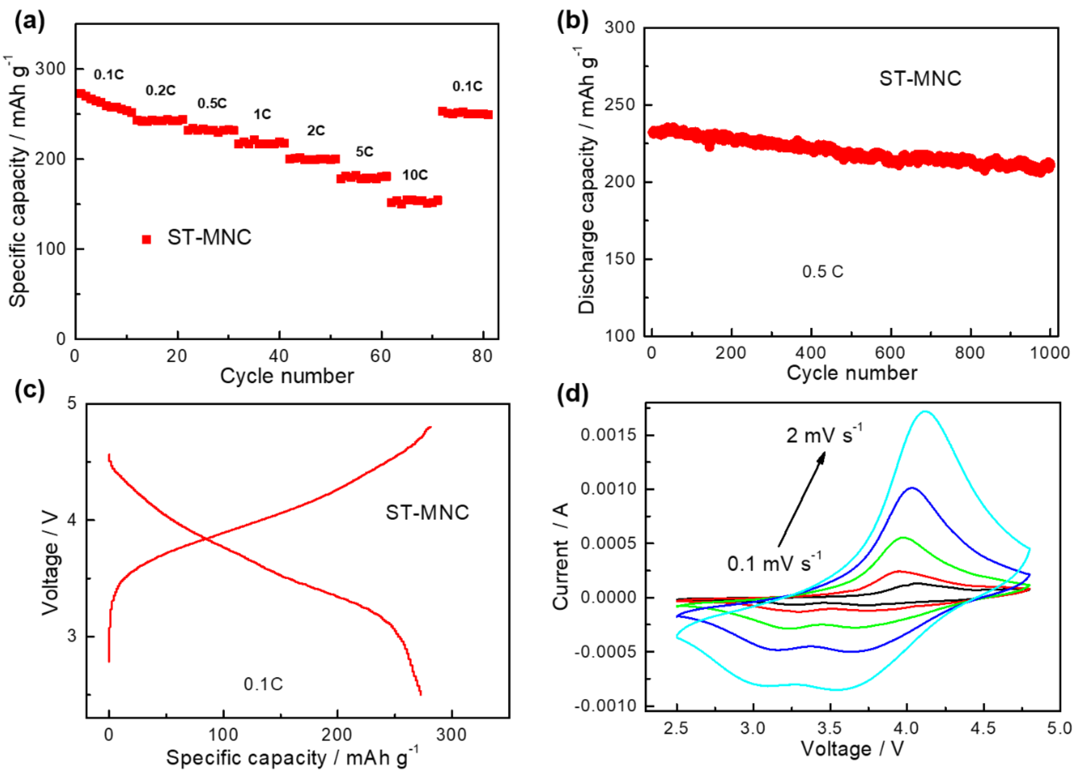

Fig. S15 Electrochemical performance of ST-MNC: (**a**) rate performance, (**b**) cycle performance and (**c**) charge/discharge profile at 0.1C; (**d**) CV curves of MNC

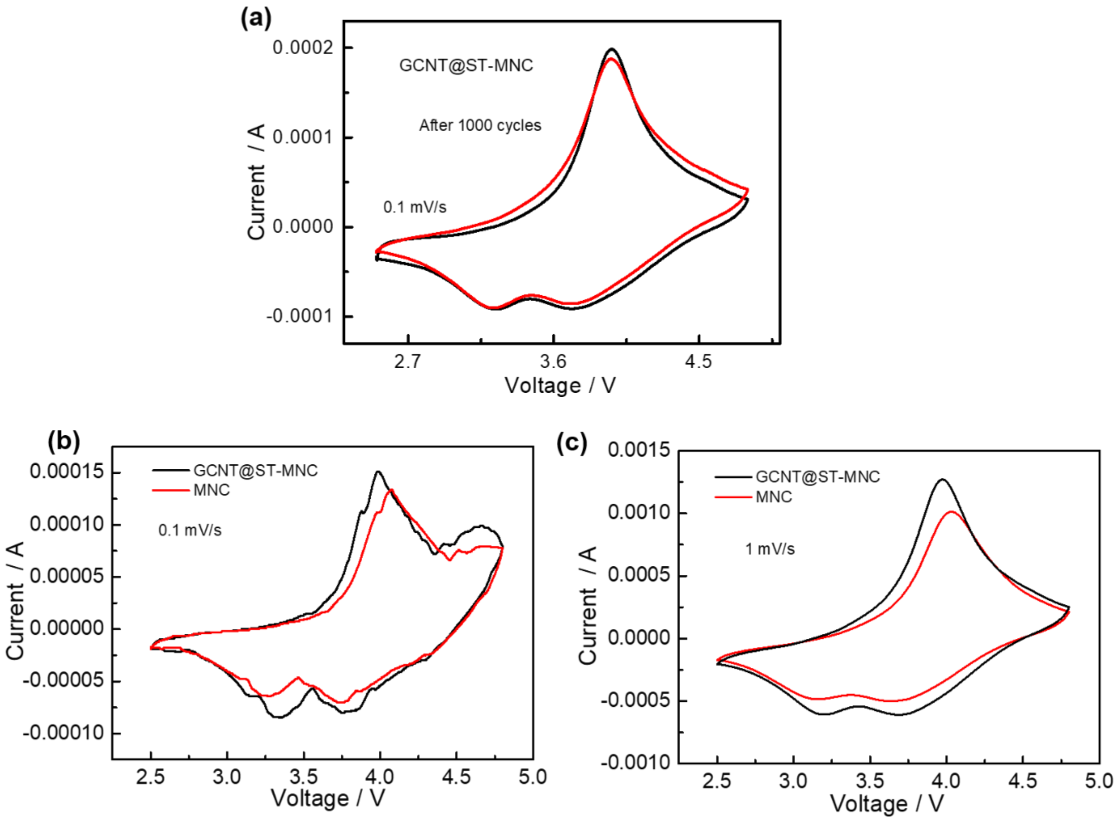

Fig. S16 CV curves of GCNT@ST-MNC after 1000 cycles (**a**). CV curve comparison of MNC and GCNT@MNC at scan rates of 0.1mV/s (**b**) and 1mV s^{-1} (**c**)

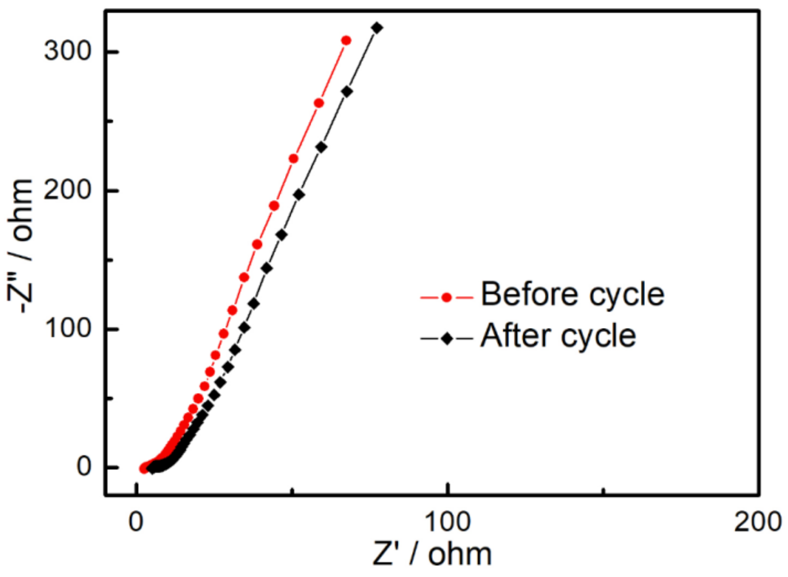

Fig. S17 EIS measurements of full cell before and after 1000 cycles

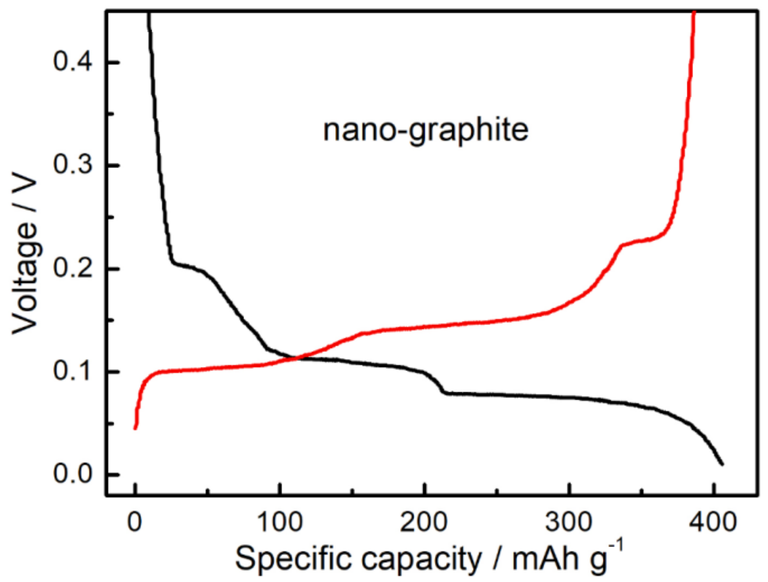

Fig. S18 Galvanostatic charge/discharge curves of nano-graphite anode
